# Developing powerful PPIE partnerships in the design of an inclusive weight management service: a case study from the NewDAWN programme

**DOI:** 10.1186/s40900-025-00709-z

**Published:** 2025-10-01

**Authors:** Caroline Mitchell, Kate Fryer, Carolyn Newbert, Jack Joyce, Susan Jebb, Paul Aveyard, Nicola Guess

**Affiliations:** 1 Academic Unit of Primary Medical Care, Faculty of Medicine, University of Sheffield, Sheffield, S10 2TN UK; 2 Expert by Experience, Oxford, UK; 3 Nuffield Department of Primary Care Health Sciences, University of Oxford, Oxford, OX2 6GG UK

## Abstract

Weight loss programmes can help people achieve remission of type 2 diabetes (T2D). People from underserved communities are less likely to participate in—and may experience poorer outcomes from—weight loss programmes than people from healthier populations already better served by healthcare systems. Unless health services including weight management programmes are based on the needs of the people most in need of them, health inequalities may get worse. Here we describe how PPIE—with a specific focus on reaching the most underserved communities—has shaped a new weight management service for T2D remission and impacted the study design, stimulating new ideas to further engagement and enhance outcomes from remission programmes more broadly. We assess our approach against the UK Standards for Public Involvement in Research and the Diabetes UK Addressing Health Inequalities in Diabetes Through Research guidance, and reflect upon achievements and areas for further development.

## Abstract (plain language summary)

Clinical research aims to help develop new services for patients or improve existing ones. However, the issues that are researched tend to be those that researchers think are important, not necessarily those patients think are important. In addition, people most in need of healthcare are those who are less likely to take part in clinical research. For example, people from Black and South Asian communities are most at risk of having type 2 diabetes, but they are less likely to take part in type 2 diabetes trials. Involving communities in research can help solve these problems. It takes time to build trust, and a genuine dialogue. In this article we describe how patient and public involvement and engagement (PPIE) has helped shape a new weight loss service for type 2 diabetes remission. We also highlight how listening in a genuine and open way can help produce new ideas for research which could lead to wider improvements in clinical care.

## Background

### The need for the NewDAWN service

Type 2 diabetes (T2D) has, until recently, been managed as a lifelong chronic disease. However, in the UK, there is now a national diabetes remission service called Path to Remission (PtR), which aims to support people to lose weight and thereby achieve remission. It offers total diet replacement (TDR), meaning eating only diet replacement products providing about 800 kcal/day for 12 weeks followed by 9 months of support to transition back to normal healthy food. The programme is very successful in the short term, with about half of people achieving remission at 1 year if they lose 10 kg or more in weight [1], though the effectiveness of the programme diminishes over time [2].

However, most people who are eligible do not join the programme and around a third cannot tolerate the programme and drop out [1, 3].

The likelihood of achieving remission from diabetes has a linear association with weight lost [1], so any programme which can achieve ~ 10 kg weight loss is likely to give participants about a 50% chance of achieving remission. We developed a research programme called NewDAWN that aimed to:

- Engage most people with diabetes diagnosed within 6 years in a programme to achieve remission through weight loss.
- Offer alternative approaches to TDR for people for whom TDR is unacceptable.
- Engage people who try one weight loss programme but do not find it helpful to remain in the NewDAWN service and try another

It is clear from these aims that to design an effective service we need to understand people’s preconceptions and past experiences about weight loss, their views about TDR programmes, and to engage with common feelings of failure that arise because people perceive that they have failed with weight loss [4] and to try to understand people’s reasons for not taking up the offer of a weight management programme. Thus, patient engagement in designing our NewDAWN service is crucial to achieving its aims and we describe our approach in this paper.

### Our PPIE approach

Encouragingly, patient and public involvement and engagement (PPIE) is increasingly common, but a closer examination of the nature of PPIE in many studies reveals that it tends to happen from the point of study design onwards, and that patients and the public are not involved in any genuine way in priority setting [5]. In nationwide PPIE work, T2D remission was found as the number one research priority for people living with T2D, amongst both the general population and ethnic minority groups [6, 7], and NewDAWN was planned based on the understanding that research in this area was a priority for people living with the condition.

In line with best practice [5, 8] PPIE has been embedded in NewDAWN throughout the planning (grant application), development work, and ongoing as we move to the trial stage.

Here, we will describe the changes made to our project as a result of PPIE, and the ways in which we let the PPIE members know how they had helped us. We then discuss how our work has aligned with national recommendations for PPIE involvement, and reflect upon successes and areas for improvement. The UK standards for public involvement in research [9] and the Diabetes UK: Addressing health inequalities in diabetes through research recommendations [10] serve as useful benchmarks against which to assess our approach.

Our PPI groups tell us that they want to know that their views have been heard and used. A central ethos of our work therefore is “you said, we did”, and we present our PPI input and our actions using this format here which supports an iterative action feedback loop throughout the research cycle in addition to following the 6 NIHR standards for PPIE [8].

1. Planning the programme grant application (Table 1).

**Table 1:** *You said, we did* during grant application planning

Grant planning	
What people said	What we did
All attendees reported having previously tried to lose weight but described the experience as unsuccessful and felt low afterwards, except one person who achieved remission. The participants responded positively to reframing the stigma of weight-loss failure and welcomed self-experimentation to find the ‘right’ programme	This reinforced our sense that this intervention was needed. All attendees with T2D felt motivated to achieve remission through dietary interventions and wanted personalisation to ‘make it work’
Attendees of South-Asian ethnicity reported that they would be more engaged with the programme if dietary advice was adapted to their cultural background	As part of a planned scoping exercise on available weight loss programmes, we looked for and included programmes which could be adapted for different cultural groups
Our contributors believed that it would be useful to have continuing contact through their weight loss attempt, and ideally this would be a health professional who had achieved remission from diabetes, though they recognised this was unlikely to be possible	We designed the NewDAWN service to have 2-weekly check-ins initially to reinforce the notion of experimentation and switching track if needed to avoid demotivation
Participants strongly favoured in-person recruitment. They reported that letters from the practice can ‘end up in the bin’. Our participants suggested using community groups to increase recruitment from underserved populations, including prison groups, support groups for people with disabilities, and community centres	Our trial will recruit participants via an in-person or telephone discussion of the possibility of remission. Given the specific inclusion criteria for the trial, we did not think that recruiting via community centres or religious groups was a “fair” way of recruiting (people may hear about it and want to take part, but ultimately not be eligible. Nevertheless, as we track recruitment, if we do not succeed in recruiting underserved communities, we will reconsider these strategies
The PPIE groups reinforced the need to maximise uptake and engagement for a wider range of underserved minority groups, to increase the applicability of the programme at a population level	We agreed to explore the impact of these issues on weight loss programme choice through a planned discrete choice experiment and qualitative work
Our PPIE contributors suggested a range of further networks that they have found to be important to them and their communities for hearing about projects, and that they would be keen to help us engage with as part of our dissemination strategy. Their suggestions ranged from local and regional charities (for example, to engage with people with learning disabilities), reputable organisations such as the Royal National Institute of Blind People (RNIB) and local groups such as the Oxfordshire Association for the Blind (our contributors highlighted that people with diabetes often have visual impairment and may require different methods of dissemination e.g. local radio stations and recorded information), and community champions who engage with their specific local communities (for example, local community café ventures nested within the local West Indian and Caribbean communities)	Each of these suggestions was implemented into the dissemination plan in the grant application which was ultimately successful. As we come to dissemination we will implement these strategies

In seeking out PPIE in the planning of NewDAWN, we took a purposive approach—we recognised that the longstanding PPIE panel was based in Oxfordshire, a relatively affluent area of the UK, albeit with some pockets of deprivation. We also recognised that teams can become set in their ways of working, and that this project would benefit from the expertise of others with more experience in community engagement and PPIE. Furthermore, People in deprived areas and from minority ethnic groups may be less likely to take up the offer of, or start a weight management programme, including PtR [3, 11]. Early data from PtR also shows that people from the lowest quartile of deprivation are less likely to complete the programme, and people from Black and South Asian ethnic groups lose significantly lower percentages of their baseline weight compared to those of White ethnicity [3]. Therefore, it was critical to involve people from these underserved communities in the development of the NewDAWN service to understand how we can best design the service to meet their needs. We therefore approached experts within the 'Deep End ' clinical research practice network [12] in the North of England to support us in identifying PPIE members from more ethnically and socially diverse backgrounds, and to advise on community engagement, involving and recruiting people from underserved backgrounds.

CN was a co-applicant and is now co-investigator on the programme. She has lived experience of T2D, weight loss and subsequent remission and was a lay member of Diabetes UK research steering group for T2D. CN has been part of the team from the initiation of the NewDAWN programme and has ensured the patient voice is heard throughout. She helps lead the patient advisory group (PAG). CN helped develop the interview questions and also carried out the interviews in partnership with JJ.

In addition, we held four focus groups and individual interviews with people with lived experience of T2D and weight loss attempts, and individual interviews aimed to gain in-depth feedback from people of South-Asian ethnicity in the study design.

The actions that we took as a result of this early PPIE input were sent back to the contributors via the PPIE panel newsletter.

2. Overseeing the NewDAWN programme (Table 2).

**Table 2:** *You said, we did* from our patient advisory group (PAG)

Input from patient advisory group (PAG)	
What people said	What we did
What will happen to people in the NewDAWN service if they drop out of one programme -is there a delay before they can start the next and how will you handle that?	Information on what to expect on being referred to each weight loss programme will be included as part of the aide memoir for the hub coaches
What about people with T2D of long duration, what options are available for them?	This input led to further research ideas (see Further Research section)
What about people who are slim with T2D, what options do they have?	
How do you convince people to try a weight loss programme when they might have tried many weight loss diets before?	Highlight the different features of weight loss programmes in the aide memoire
How do you encourage people to try TDR since people might see it as a “crash/fad diet”	Explain it’s an NHS-approved programme

**Fig. 1:**
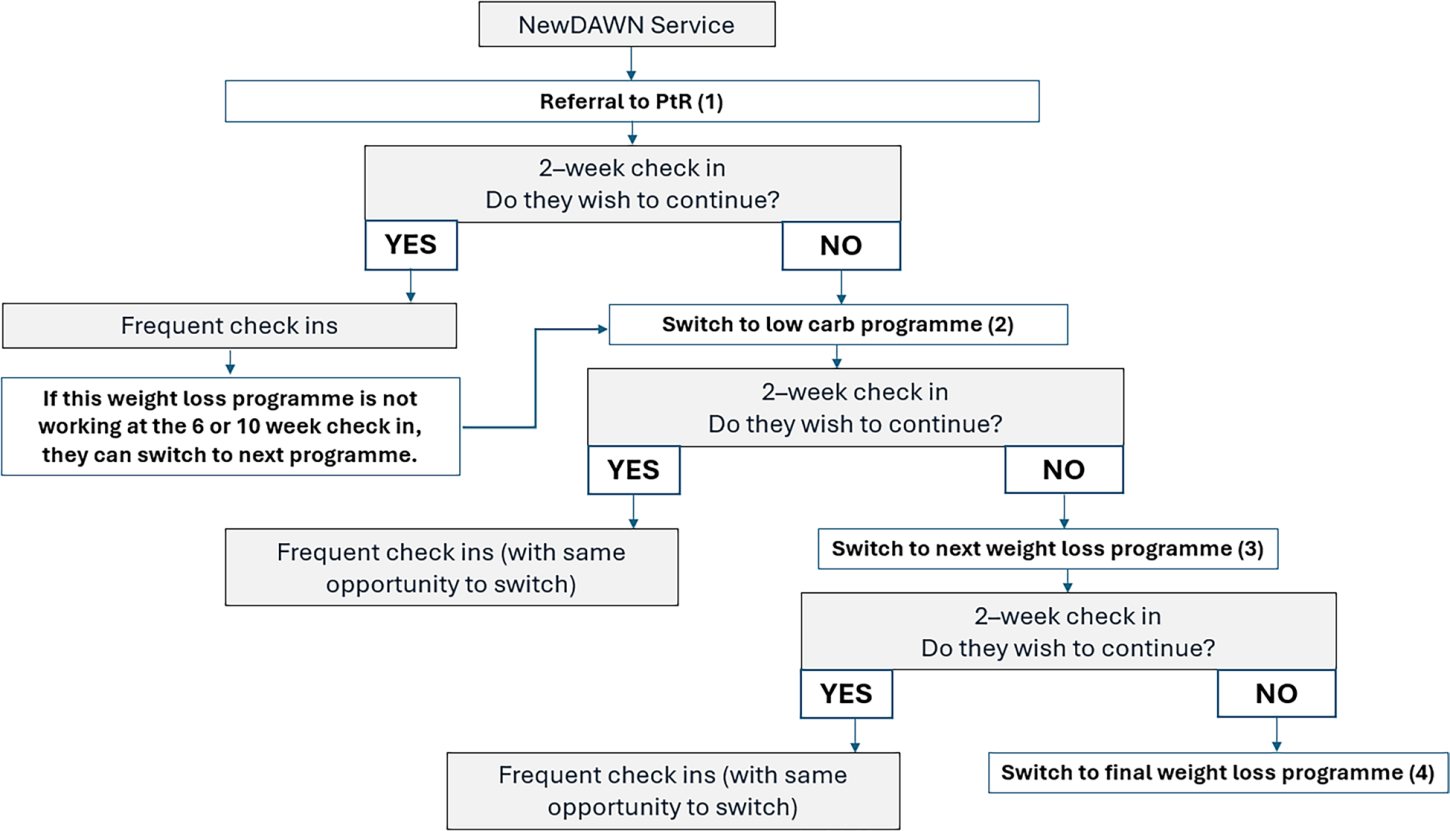
An Overview of the NewDAWN service

The initial PPIE informed the work of our academic team to design the skeleton of the NewDAWN service, which is shown in Fig. 1, and the programme of work was awarded an NIHR programme grant in 2021 (NIHR202860). The service is described in depth elsewhere [13]. Briefly, people newly diagnosed with T2D will be referred by their GP to the NewDAWN hub, staffed by generalist health coaches, who will support the participant in experimenting with up to four weight loss programmes.

To oversee the programme which comprises development work, piloting, main trial and cost-effectiveness analysis we set up a patient advisory group (PAG) of 6 people living with T2D during the first year of development work. To date, the PAG has provided input into the design of NewDAWN, and identified factors we needed to consider in overall planning.

3. Designing the NewDAWN service.

The innovation of the NewDAWN is to combine different types of weight loss programmes into a single service within primary care. Service design and streamlining were the issues on which we focused our PPIE activities from the earliest stages of our research.

The key aspects of the design of the NewDAWN service were:

1. Which weight loss programmes to offer?
2. How to give all participants an equitable chance of weight loss?
3. Whether the design needed to be different for different population groups?

### ***Which weight loss programmes to offer? (***Table 3***)***

**Table 3:** *You said, we did* in deciding which weight loss programmes to offer

Which weight loss programmes to offer	
What people said	What we did
People mentioned the following different characteristics of weight loss programmes that they stated they had experienced and had liked or that they thought would make a weight loss programme appealing to them: Social/companionship/camaraderie Being able to come off meds/”not having diabetes anymore” Support/Advice, especially outside of sessions (eg WhatsApp) Having people “’like me” in the group Focus on behaviours, not weight Long-term behaviour change, (not crash diet). Flexibility, e.g., being able to have two MR, then main meal Food-based—just smaller portions	In combination with the lack of intensive weight loss programmes available on the NHS or from local authorities, this confirmed our hypothesis that the development of a food-based low-cal (about 800 kcal per day) would be essential to the success of NewDAWN. The qualitative research in DIAMOND highlighted a preference for remote options This led us to develop the idea of using a remote version of a previously developed DIAMOND intervention / programme as the second option in the NewDAWN service
	This work also prompted us to aim to have four weight loss programmes which differed in one or more of these characteristics to the extent possible, ie having one that was group-based; one focused on healthy behaviours, not just weight
	In recognition that some groups (white males and south Asian females) preferred to have attend groups with “people like me”, we explored whether there was a need for further research in these areas (final section)

We carried out open interviews, focus groups and discussions on the idea for NewDAWN. We briefly described the NewDAWN service, and the idea of including different weight loss interventions. We then asked people to tell us their experiences of managing their diabetes and any weight loss interventions for T2D they had tried. Finally, we asked the specific question “what attributes or characteristics would you like to see that would encourage you to give that weight loss programme a go?”.

In total we carried out 14 one-to-one interviews (Gender: 8 men, 6 women; Ethnicity: 7 South Asian, 2 African-Caribbean, 5 White British), 2 focus group discussions with community groups in diverse urban areas with one group comprised of members from the African-Caribbean community (31 people in total) and one informal discussion at a local PPIE event (Diversity in Research—12 people).

We updated all our PPIE members with a one-page summary to let them know how they had changed our research methods.

### Aiming to give all participants an equitable chance of success

The development work of NewDAWN included a qualitative work package to understand (1) how to encourage uptake and adherence of the NewDAWN programme, and (2) how to reframe ‘failure’ if a programme does not work for an individual. This involved qualitative interviews with people living with T2D and health coaches delivering existing diabetes prevention and remission services [13]. The guiding principle of the development work was to ensure voices from underserved communities are central to our research and that people from those communities trusted and felt support to take part in research. The information collected from this work package was critical in informing the aide memoire/script for the coaches who would be delivering the NewDAWN intervention.

**Fig. 2:**
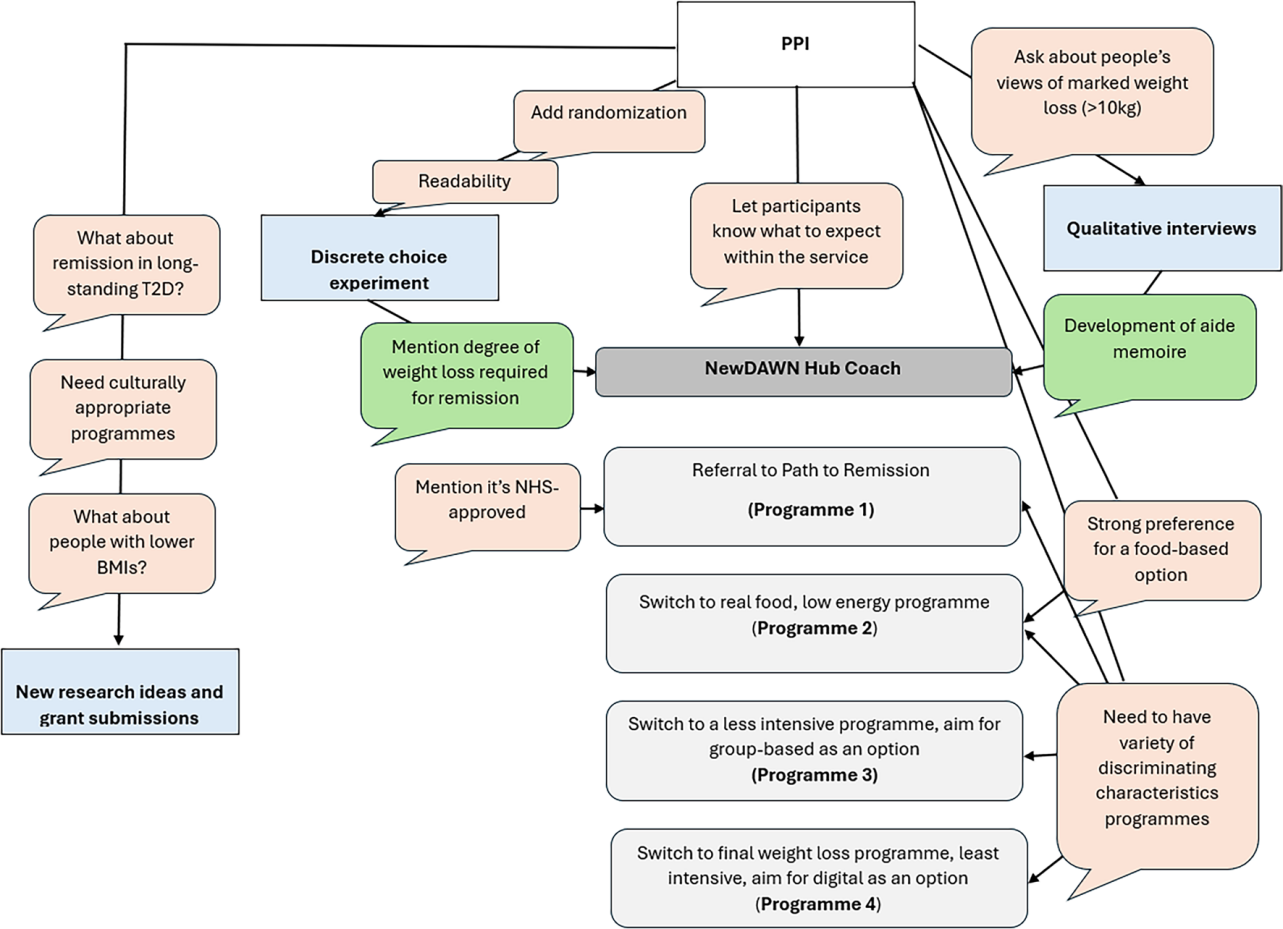
A schematic summarising the ways PPI influenced the NewDAWN programme, and also inspired further grant ideas

We assembled PPIE groups throughout this work-package, working iteratively and their input influenced our approach in multiple ways (Fig. 2):

1. Developing the interview question (Table 4).

2. Support with sampling decisions (who to reach and advice on how to reach)—we aimed for a maximum variation sample in experience living with T2D. We asked PPIE if doing something, or going somewhere would encourage involvement (Table 5).
3. Reflections on findings. We reported our themes to PPIE for their views because we wanted to make sure that the research resonated with their experience. We aimed to centre our around the lived experience of individuals with T2D and ensure that the communication resource developed from our findings would be appropriate (Table 6).

**Table 4:** *You said, we did* when developing the qualitative interview questions

Giving all participants an equitable chance of success: developing the interview question	
What people said	What we did
Our PPIE panels told us that our narrative interview question could be too narrowly focused on “big impacts” of diabetes, and possibly not be open enough to account for people who had not maintained remission. People also told us that it could be useful to re-ask the first question at the end of the interview to solicit further reflections based on their initial story	The narrative interview question (a qualitative interview wherein a single question is asked and participants given the space to tell their story) was co-developed in discussions with PPIE for what we could ask that would be meaningful for people living with T2D, and be open enough to solicit a longer answer from participants. We changed the word “impact” to “effect” which PPIE found more appropriate. We also included the initial question (reframed slightly so to avoid repetition) as a prompt to invite participants to reflect on their initial response

**Table 5:** *You said, we did* to consider sampling decisions

Giving all participants an equitable chance of success: support with sampling decisions	
You said	We did
People mentioned we could sample based on dietary limitations, including dietary preferences. PPIE members also explained that we’d be like to reach more people, who are underserved by research, by spending time at community and religious centres particularly in areas experiencing economic deprivation, such as from coastal communities	PPIE supported our decisions of what characteristics to prioritize in our sample. Their recommendation was to include ‘dietary limitations’, which we did. We sought variation in people’s reported diets. PPIE comments also meant we targeted recruitment from geographical areas of England matching what PPIE prioritized. For example, we recruited via a community advocacy organization based in the Northeast coast of England

**Table 6:** *You said, we did* to reflect on the qualitative findings from the interviews

Giving all participants an equitable chance of success: reflections on qualitative findings	
What people said	What we did
Our PPIE panels confirmed how we had interpreted our participants’ responses, and that the themes we generated were recognizable. PPIE members explained that ‘affect’ (a term we used) was potentially unclear and our suggested alternative ‘emotional’ could imply negativity. PPIE members asked whether we considered diabetes stigma as part of our analysis and what impact stigma would have had on people living with T2D	We reframed the analysis way from ‘affective’ or ‘emotive’ beginnings to a more neutral, and acceptable ‘Coming to terms with T2D’. PPIE’s request to include stigma led us to state the impact of stigma on people’s care—and in the discussion, we added context to our findings to explain how any ‘takeaways’ from the article should also include strategies to address different forms of stigma

In addition, we supported a PPIE member as a lived experience co-researcher to see the unvarnished truth of research and actively participate in doing the research. This is a participatory approach with research done *with* or *by* the public rather than *about* or *for* them [14]. As well as being a part of the aforementioned PPIE development and reflections, the co-researcher also led interviews with the support of JJ.

Subsequent PPIE and co-researcher involvement included translating the findings from the development work into the NewDAWN remission communication intervention (Fig. 2), aiming to ensure that that the communication in and as part of the NewDAWN trial is relevant, appropriate, acceptable and gives all people living with T2D the best chance possible at taking up the prospect of remission.

### ***Whether the design needed to be different for different population groups (***Table 7***)***

**Table 7:** *You said, we did* when designing the discrete choice experiment (DCE)

Whether the discrete choice experiment (DCE) design needed to be different for different population groups	
What people said	What we did
Nobody in our PPIE group mentioned rapid weight loss or very marked weight loss (eg > 10 kg) as an important characteristic. We thought this was important given that the degree of weight loss is the primary determinant of remission, and early (rapid) weight loss is a good predictor of overall weight loss	We therefore modified the DCE protocol to randomize half the sample to read a short statement about the health benefits (including T2D remission) of large amounts of weight loss (~ 10 kg) and half would not get the statement. The goal of this randomization was to understand whether one of the aspects of the hub coaching would be to explain the importance of marked weight loss in achieving remission
Some PPIE members felt that intermittent “fasting” needed to be explained as many people might view it as a religious practice and not something done under medication supervision for the purposes of weight loss	We also used the feedback from our PPIE groups to modify the wording in the DCE to ensure it was understood by a diverse population
The PPIE members thought that “macronutrient-based diets”, like low-carbohydrate was confusing, and instead should use the words “food-based” and give examples	The wording was changed accordingly on the DCE

Our planned NewDAWN work included a discrete choice experiment to help us identify which weight loss programmes we should include in the NewDAWN service based on the reported respondents of the experiment. However, following a scoping review which identified a limited number of effective weight loss interventions that were available that we could refer to, this aim was rendered moot. However, we were able to use the large sample size of the DCE (discrete choice experiment) (n = 4000) to try to understand whether different populations had meaningfully different preferences for weight loss programmes and thus whether we could alter the service in such a way as to account for this.

The PPIE we carried out helped us to modify the design of the DCE in several ways:

The DCE [15] found few differences in weight loss programme preferences by gender or ethnicity which was reassuring given the limited choice of programmes available to us. However, individuals from ethnic minority populations identified more with programs where others shared their characteristics. This, alongside poorer engagement with and outcomes from weight loss programmes amongst minority ethnic groups prompted us to expand our research (see filling research gaps section). 

### Filling research gaps

One of the most important aspects of PPIE is that participants in these groups will often raise queries which are outside of the scope of the research project.

We have listened to these views and taken action (Fig. 2 and Table 8):

**Table 8:** *You said, we did* to fill research gaps

Filling research gaps	
What people said	What we did
Our PPIE members wanted options for programmes to manage T2D that didn't just require weight loss	We submitted a programme grant on a low-carbohydrate intervention—which does not specifically aim for weight loss—on T2D management in primary care which moved to stage 2
Our groups also raised a concern that many of the weight loss programmes they had tried were not suitable for people from different cultural backgrounds	Members of our team secured a programme development grant (PDG) to develop community partnerships with Black and South Asian communities to help design T2D remission services
Many of our groups had had T2D for more than 6 years, and are therefore not eligible for PtR or the NewDAWN service. They asked us to explore and work on programmes that could help people who have T2D of long duration also be able to come off medications and achieve remission	We are developing research ideas for people with T2D of long durations

## Discussion

In 2019 NIHR released a set of six standards for public involvement in research [9], to help researchers and organisations improve the quality and consistency of public involvement in health and care research (Table 9).

**Table 9:** The NIHR 6 standards for PPIE

Inclusive opportunities	Public involvement partnerships are accessible and include a range of people and groups, as informed by community and research needs
Working together	Work together in a way that values all contributions, and that builds and sustains mutually respectful and productive relationships
Support and learning	Offer and promote support and learning opportunities that build confidence and skills for public involvement in research
Communications	Use plain language for well-timed and relevant communications, as part of involvement plans and activities
Impact	Seek improvement by identifying and sharing the difference that public involvement makes to research
Governance	Involve the public in research management, regulation, leadership and decision making

These align with recommendations from Diabetes UK [10] to consider how we can “improve patient and public involvement and engagement to make diabetes research more inclusive of and relevant to diverse communities” and “improve research design so that the people who could benefit most are represented”.

Our PPIE work has aimed to offer *inclusive opportunities* and ensure that “public involvement partnerships are accessible and include a range of people and groups (as informed by community and research needs) be involved in research”. For example, we attended events run by underserved communities to discuss weight loss programmes and T2D remission, and following Diabetes UK guidance have aimed to oversample people from underserved communities in our PPIE groups and qualitative work.

Our DeepEND collaborators have also re-iterated how trust is built over long periods of time. By updating—and giving back to—our contributors we hope to sustain a long-term relationship to enable our panels to continue contributing to research in the long term [16], including in setting the research priorities Others have also noted the need to challenge the typical patient involvement paradigm whereby patients and the public are only involved to a tokenistic extent, and PPIE rarely influences the research agenda [5]. We also made clear to participants that even when we could not act upon their suggestions immediately, that we would aim to explore their suggestions via other avenues. For example, our PPIE groups felt that there is a lack of culturally appropriate remission programmes. Members of our group submitted, and have been awarded, a programme development grant (PDG) —with a community group lead as a co-principal investigator—entitled: “From consultation to citizen control: Ensuring no one is left behind in T2D remission research”. It is inspired by Arnstein’s ladder of citizen participation [17], and aims to put the power back in the hands of the communities most affected by T2D. Involving patients and the public in *governance of research management, regulation, leadership and decision making* takes time, and we view our PDG as the first step in this process.“You said, we did” is also part of our commitment to *value all contributions* and sustain *mutually respectful and productive relationships*. One of the most important lessons from our PPIE contributors is to ensure communication is a two-way street. We have aimed to keep our PAG and PPIE members updated so they feel included in the research process. For example, in many trials there can be an inactive period while waiting for governance approvals before research activities can start. While it may not have been appropriate for a full patient advisory group (PAG) meeting at these times, our lay co-investigator let us know that people value being updated. So, we send regular email updates on our progress between meetings. We have aimed to ensure we communicate using plain language these communications, and aim to plan ahead to let our contributors know of further opportunities to become involved.

We have offered *support and learning* to our lay co-investigator (CN) who has received one-day’s bespoke training in developing interview questions at the University of Oxford. She was also supported by an experienced qualitative researcher (JJ) who observed the interviews and carried out ‘debriefing’ meetings where the lay co-investigator and qualitative researcher reflected on how the interview went (both in terms of approach and how they interpreted the response to the question from their perspective).

Feedback from JJ indicated that involvement of CN in the interviews enabled the participants to open up more fully, and establish a rapport more quickly. CN reflects that people were reassured by her understanding their concerns, by not having to explain things that might have been difficult to go into detail about [18]. CN was also able to pick up on things in the interviews that JJ acknowledges he would have missed—e.g. things that were noticeably absent in responses (that I did not know were absent because I don’t have T2D). The cumulative effect of this was that interviews yielded more rich information, and information power was reached more quickly. However, this is an area which we can build on with our other PPI input including our PAG. At present, the relationships with our PPIE contributors are relatively new. As we continue our dialogue throughout this 5-year project we hope to be able to offer more substantive involvement in research and will offer training opportunities where appropriate. This aligns with Diabetes UK’s recommendation to ensure the research community is representative of the general population [10]. Many of our contributors are passionate about improving care for people with T2D and are eager to be involved in dissemination. We envisage offering training opportunities that build confidence and skills to enable them to deliver talks at conferences alongside our academic team. 

Despite the efforts we have made to involve patients and the public in our NewDAWN project, the outcome that matters is whether we succeed in recruiting a greater proportion of people from underserved communities, and whether these groups benefit from the service in the same way as people from White and less economically deprived communities. We will report engagement with and outcomes by ethnic group and by socioeconomic status (IMD) from our trial. We will also carry out a process evaluation to understand differences in uptake and outcomes including interviewing practice staff from more ethnically-diverse and economically-deprived catchment areas, and patients themselves, including those who do and don’t take up an invitation to participate. Our ethnically diverse PAG group and PPIE contributors are keen to be involved in dissemination of our findings and we will need to consider effective strategies for dissemination of our findings among under-served groups, as recommended by Diabetes UK [10].

We also emphasise that the NewDAWN service is an adaptive service. Therefore, while we acknowledge that at present, there is a lack of weight loss programmes developed with and for people from minority ethnic communities, our PDG and subsequent work aims to develop and test such programmes. Hence, if we or others generate evidence for any weight loss programme, it could be slotted into the NewDAWN service as an option.

## Conclusions

We are appreciative of the benefits of working with patients and the public, and are proud to be able to continue areas of research they have suggested are important. This output aims to ensure that the ‘Impact’ of our PPIE work does not just improve the research that we have been able to do; by describing our experiences in this article, we hope to share the difference that public involvement can make to research and clinical service design. 

## Data Availability

Not applicable.
